# Interferon lambda protects the female reproductive tract against Zika virus infection

**DOI:** 10.1038/s41467-018-07993-2

**Published:** 2019-01-17

**Authors:** Elizabeth A. Caine, Suzanne M. Scheaffer, Nitin Arora, Konstantin Zaitsev, Maxim N. Artyomov, Carolyn B. Coyne, Kelle H. Moley, Michael S. Diamond

**Affiliations:** 1 Departments of Medicine, Washington University School of Medicine, Saint Louis, MO 63110 USA; 2 Obstetrics and Gynecology, Washington University School of Medicine, Saint Louis, MO 63110 USA; 3 Departments of Pediatrics, University of Pittsburgh School of Medicine, Pittsburgh, PA 15224 USA; 4 The Center for Microbial Pathogenesis, Children’s Hospital, University of Pittsburgh School of Medicine, Pittsburgh, PA 15224 USA; 5 Department of Pathology and Immunology, Washington University School of Medicine, Saint Louis, MO 63110 USA; 6 Computer Technologies Department, ITMO University, St. Petersburg, 197101 Russia; 7 The Andrew M. and Jane M. Bursky Center for Human Immunology and Immunotherapy Programs, Washington University School of Medicine, Saint Louis, MO 63110 USA; 8 Department of Cell Biology and Physiology, Washington University School of Medicine, Saint Louis, MO 63110 USA; 9 Department of Molecular Microbiology, Washington University School of Medicine, Saint Louis, MO 63110 USA; 10 Present Address: Department of Pediatrics, The University of Alabama at Birmingham, Birmingham, AL 35233 USA

## Abstract

Although Zika virus (ZIKV) can be transmitted sexually and cause congenital birth defects, immune control mechanisms in the female reproductive tract (FRT) are not well characterized. Here we show that treatment of primary human vaginal and cervical epithelial cells with interferon (IFN)-α/β or IFN-λ induces host defense transcriptional signatures and inhibits ZIKV infection. We also assess the effects of IFNs on intravaginal infection of the FRT using ovariectomized mice treated with reproductive hormones. We find that mice receiving estradiol are protected against intravaginal ZIKV infection, independently of IFN-α/β or IFN-λ signaling. In contrast, mice lacking IFN-λ signaling sustain greater FRT infection when progesterone is administered. Exogenous IFN-λ treatment confers an antiviral effect when mice receive both estradiol and progesterone, but not progesterone alone. Our results identify a hormonal stage-dependent role for IFN-λ in controlling ZIKV infection in the FRT and suggest a path for minimizing sexual transmission of ZIKV in women.

## Abstract (web summary)

Zika virus infections can cause devastating congenital birth defects but the underlying interactions with the host immune system are not well understood. Here, the authors examine the immune basis of vaginal protection and susceptibility to Zika viral infection, and identify a hormonal dependent role for interferon-lambda-mediated protection against disease.

## Introduction

Zika virus (ZIKV) is an emerging mosquito-transmitted flavivirus that can cause congenital birth defects during infection of pregnant women, non-human primates (NHP), and mice^1–7^. Unlike other flaviviruses, ZIKV can be transmitted sexually in addition to by mosquito inoculation^8–11^. Recent studies suggest that sexual transmission of ZIKV may be underestimated^12^, and its consequences to the female reproductive tract (FRT) have not been extensively evaluated. In comparison, ZIKV infection can persist in the testes, seminal fluid, and sperm, which is associated with oligospermia in humans^2,13^ and testicular atrophy and decreased fertility in mice^1,2,14–16^. Less is known about which cell types ZIKV targets in the FRT, although studies in mice and NHPs indicate that sexual exposure leads to productive infection of the vagina^8,17–20^

In women, the vaginal epithelium is the first line of defense against sexually transmitted diseases. The menstrual cycle is regulated hormonally to optimize conditions for ovulation, implantation, and conception. As part of this process, the thickness and components of the epithelial barrier in the vagina vary through stages of the estrous cycle in mice and menstrual cycle in humans^21,22^. In mice, this results in resistance to intravaginal infection with ZIKV or herpes simplex virus (HSV) during the estradiol-high, estrous phase but susceptibility during the progesterone-high, diestrous phase^18,23,24^. The mechanistic basis for these hormonal stage-dependent changes in susceptibility to infection remains largely unknown.

The innate immune response acts as a barrier against sexually transmitted diseases. One study reported that interferon (IFN)-ε, a type I IFN subtype that signals through Ifnar1/Ifnar2 heterodimers^25^, is constitutively expressed by epithelial cells in the uterus, cervix, vagina, and ovary and protects the FRT of mice from intravaginal infection with HSV^26^. However, the role of IFN-ε in other viral infections, or in humans, remains uncertain. For example, ZIKV replicates in the vaginal mucosa of mice with minimal induction of antiviral type I IFN responses^27^. This latter study also reported that intravaginal treatment with acitretin, a highly teratogenic retinoic acid derivative^28^, induced type I (α/β) and III (λ) IFNs and resulted in diminished ZIKV infection. Type III IFN-λ is an antiviral cytokine that functions at barrier surfaces and is induced downstream of pathogen recognition receptor sensing and activation of mitochondrial antiviral signaling protein (MAVS)^29^. IFN-λ binds to and signals through selectively expressed heterodimeric receptors (Ifnlr1/Il10rβ), which distinguishes it from type I IFNs that bind to the more broadly expressed Ifnar1/Ifnar2 heterodimers. IFN-λ has antiviral functions against ZIKV in the context of infection of the maternally derived decidua and fetally derived placenta during pregnancy in mice and humans^30–32^.

Here, we evaluate ZIKV infection and host responses in the tissues of the FRT. Initial experiments in primary human vaginal and cervical epithelial cells demonstrate that IFN-β or IFN-λ treatment induces transcriptional programs that inhibit ZIKV infection. By performing studies in hormone-synchronized, non-pregnant, ovariectomized (OVX) female mice, we confirm resistance to intravaginal ZIKV infection during the estradiol-high, estrous phase and susceptibility during the progesterone-high, diestrous phase^17,18^. As mice lacking type I IFN signaling responses are resistant to intravaginal ZIKV infection during the estrous phase, IFN-ε likely is not the dominant contributing factor to protection during the estrous phase of the reproductive cycle. As ZIKV replicates to higher levels in the vaginal and cervical epithelial layers of progesterone-treated *Ifnlr1*^−/−^ than wild-type (WT) mice, these data suggest that IFN-λ signaling contributes to the antiviral response in the FRT in the diestrous phase. However, *Ifnlr1*^−/−^and WT mice sustain similar levels of ZIKV infection in FRT tissues when animals were treated with estradiol and progesterone (proestrous phase, which corresponds with the hormone profile in the luteal phase of humans), suggesting a hormonal stage-dependent effect of IFN-λ. Consistent with the observation, IFN-λ is not induced after ZIKV infection in mice treated with estradiol and progesterone. Nonetheless, when pegylated IFN-λ2 is administered to mice treated with estradiol and progesterone, markedly diminished ZIKV infection is observed in the vagina, cervix, uterus, and other distant sites. Collectively, these results provide insights into the tissue tropism of ZIKV in the FRT, the effects of sex hormones on ZIKV infectivity, and the innate immune response to ZIKV in the vagina.

## Results

### Effects of IFN-β and -λ on human vaginal and cervical cells

**Fig. 1:**
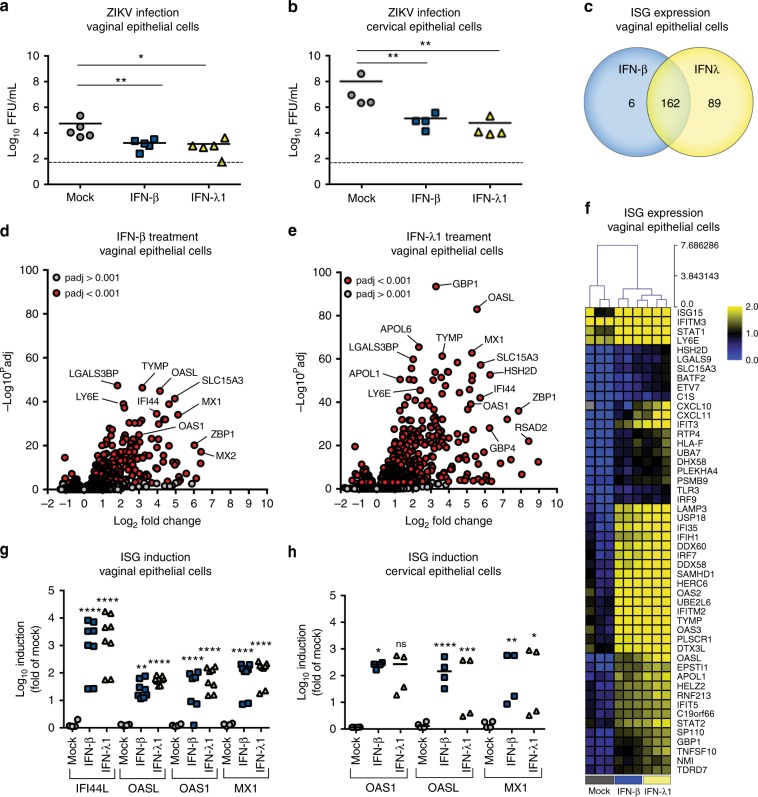
IFN-λ restricts ZIKV infection of primary human vaginal and cervical epithelial cells. ZIKV infection titers from four preparations of primary human vaginal epithelial cells (HVECs, **a**) and three preparations of primary human cervical epithelial cells (HCECs, **b**) treated with medium (mock) or 100 ng/mL of IFN-β or IFN-λ1 (~16 h) and then infected with ZIKV (Paraiba 2015) for 48 h. Note, one of the biological preparations of HVECs was interrogated in two separate experiments. Solid lines indicate mean values, and dotted lines denote limit of detection. **c** Venn diagram of transcripts differentially expressed by IFN-β or IFN-λ1 treatment of HVEC as assessed by RNAseq. **d**, **e** Volcano plots of HVEC treated with IFN-β (**d**) or IFN-λ1 (**e**) denoting ISGs (red circles) differentially expressed by treatment (*P* < 0.001, as determined using the DeSeq2 package in R). Transcripts that were not differentially expressed are denoted by gray circles. **f** Hierarchical clustering heat map (based on log(RPKM) values) of the top 50 ISGs induced by treatment of HVECs with IFN-β or IFN-λ1 compared to mock-treated controls. Color intensity indicates the level of gene expression (yellow for upregulation and blue for downregulation), and gray indicates that no reads were detected for that transcript. **g**, **h** Induction of representative ISGs (*IFI44L*, *OASL*, *OAS1*, and *MX1*) after treatment of HVECs (**g**) or HCECs (**h**) with 100 ng/mL of IFN-β or IFN-λ1 for 16 h as assessed by RT-qPCR. Statistical analyses in **a**, **b**, **g**, and **h** were performed using a one-way ANOVA (ns = not significant; *, *P* < 0.05 **, *P* < 0.01 ***, *P* < 0.001 ****, *P* < 0.0001)

To begin to characterize the innate immune response of FRT tissues to ZIKV infection, we first evaluated the effect of recombinant IFN-β and IFN-λ on ZIKV infection of primary human vaginal epithelial cells (HVECs) obtained from four different donors and primary human cervical epithelial cells (HCECs) obtained from three donors. Pre-treatment of HVECs or HCECs with 100 ng of IFN-β or IFN-λ1 reduced ZIKV infection by 32 to 39-fold (HVECs) (*P* < 0.05) or 695 to 1575-fold (HCECs) (*P* < 0.01) compared to mock-treated controls (Fig. 1a, b). To gain further insight into the antiviral effects of IFN treatment on HVECs, we performed whole-genome RNAseq-based transcriptional profiling of mock-, IFN-β-, or IFN-λ1-treated cells from three donors followed by differential gene expression and gene set enrichment analyses. IFN-λ1 treatment resulted in differential expression of 251 transcripts compared to 168 after IFN-β treatment (Supplementary Figure 1a, b). Of these differentially expressed transcripts, 162 were shared by IFN-β and IFN-λ1, 89 were induced uniquely by IFN-λ1, and six were induced specifically by IFN-β (Fig. 1c–f). Reverse transcriptase, quantitative polymerase chain reaction (RT-qPCR) studies confirmed the induction of a number of canonical IFN-stimulated genes (ISGs) in HVECs and HCECs treated with IFN-β or IFN-λ1 (Fig. 1g, h). Gene set enrichment and differential expression analyses of transcripts specifically modulated by IFN-λ1 treatment did not reveal any unique pathways, but demonstrated that several ISGs (e.g., *IFIT2*, *IRF1*, and *IFI30*) were induced more robustly by IFN-λ1 than IFN-β (Fig. 1f and Supplementary Figure 1c–e). Taken together, type I and type III IFNs induced a strong ISG response in human FRT cells that resulted in reduced ZIKV infection.

### ZIKV resistance during estrous phase is IFN-independent

**Fig. 2:**
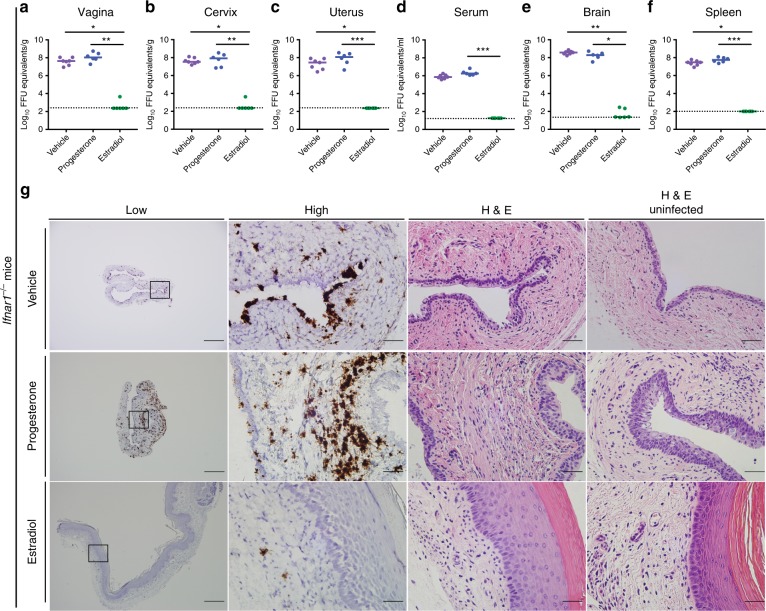
Estradiol protects *Ifnar1*^*−/−*^ mice from intravaginal ZIKV infection. Six-week-old *Ifnar1*^−/−^ OVX mice were given a hormone replacement regimen of estradiol, progesterone, or vehicle. Subsequently, mice were inoculated via intravaginal route with 10^6^ FFU of ZIKV (Dakar 41525). At 6 dpi, ZIKV RNA was measured from the vagina (**a**), cervix (**b**), uterus (**c**), serum (**d**), brain (**e**), and spleen (**f**). Dotted lines indicate the limit of detection (LOD) for each assay. Results are pooled from two or three experiments. Bars indicate median values (Kruskal–Wallis test: *, *P* < 0.05 **, *P* < 0.01 ***, *P* < 0.001). Vaginal tissue was stained using H&E or by ISH for ZIKV RNA (**g**). Low magnification scale bar = 500 μm, high magnification scale bar = 50 μm. Numbers of mice: vehicle *n* = 7, progesterone *n* = 6, estradiol *n* = 6. Representative images are shown

Our transcriptional data suggested that type I or III IFNs might confer antiviral effects in the lower FRT. Since previous studies had shown resistance to ZIKV and HSV infection during the estradiol-high, estrous phase^18,33^, we hypothesized that this might be due in part to constitutive or rapidly induced expression of antiviral IFNs. Indeed, prior data suggested that IFN-ε is expressed in the vagina during the estradiol-high, estrous phase and protected mice from HSV infection during the diestrous phase^26^. We assessed the contribution of type I IFN signaling to the antiviral effect of estradiol against intravaginal ZIKV infection using *Ifnar1*^−/−^ mice. However, estradiol-treated OVX *Ifnar1*^−/−^ mice remained resistant to intravaginal ZIKV infection. Tissue samples from the vagina, cervix, uterus, serum, brain, and spleen at 7 days post infection (dpi) had little ZIKV RNA (Fig. 2a–f). In comparison, vehicle or progesterone treatment of OVX *Ifnar1*^−/−^ mice resulted in ZIKV infection of all fluids and tissues tested. In situ hybridization (ISH) with a ZIKV-specific RNA probe in vaginal tissues from *Ifnar1*^−/−^ mice treated with vehicle or progesterone revealed infection of the epithelial layer and lamina propria, whereas animals treated with estradiol had virtually no infection present (Fig. 2g, left panels). No marked tissue injury was apparent in the vagina at 7 dpi, although a cellular infiltrate was present in progesterone-treated, infected animals (Fig. 2g, right panel).

**Fig. 3:**
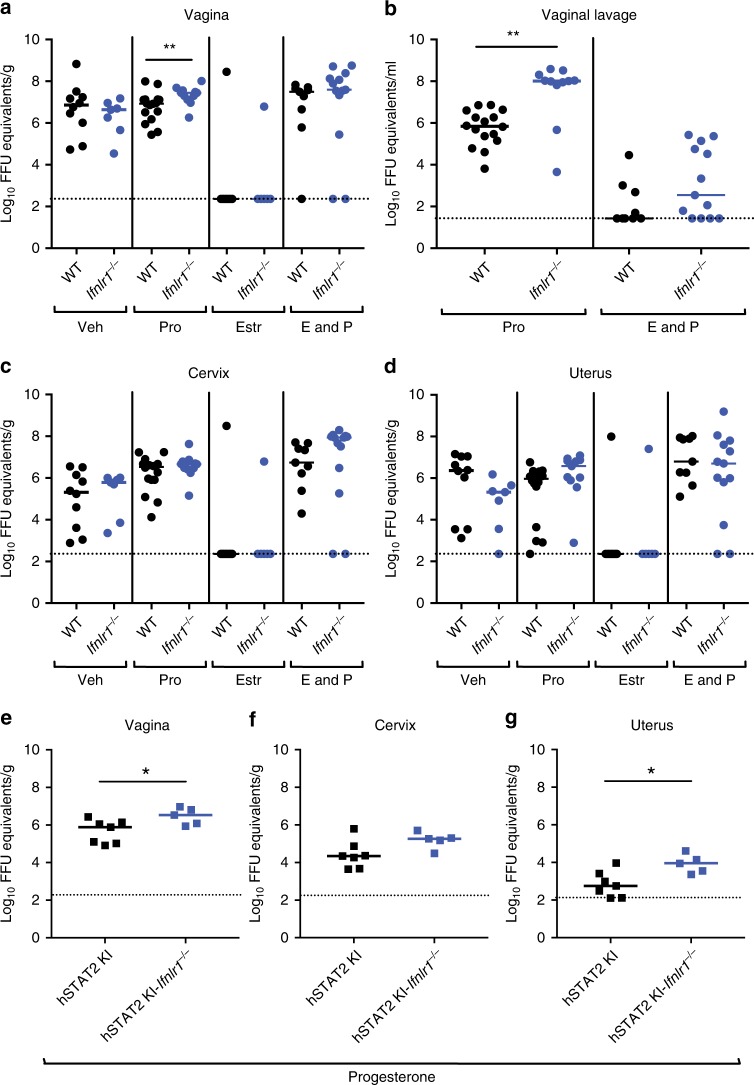
Hormone treatment modulates the severity of ZIKV infection in the vagina of OVX *Ifnlr1*^*−/−*^ and hSTAT2 KI *Ifnlr1*^*−/−*^ mice. Six-week-old WT, *Ifnlr1*^−/−^, hSTAT2 KI, or hSTAT2 KI *Ifnlr1*^*−/−*^ OVX mice were given a hormone replacement regimen of estradiol, progesterone, estradiol and progesterone, or vehicle. WT and *Ifnlr1*^*−/−*^ mice were treated with 1 mg of anti-Ifnar1 mAb at day −1. On day 0, mice were inoculated via intravaginal route with 10^6^ FFU of ZIKV (Dakar 41525). At 3 dpi, vaginal lavage was performed (**b**). At 7 dpi, ZIKV RNA was measured from the vagina (**a**, **e**), cervix (**c**, **f**), and uterus (**d**, **g**). Dotted lines indicate the LOD. Results are pooled from two or three experiments. Bars indicate median values (Mann–Whitney test: (*, *P* < 0.05 **, *P* < 0.01). Numbers of mice: Vehicle: WT + anti-Ifnar1, *n* = 10; *Ifnlr1*^−/−^ + anti-Ifnar1, *n* = 7. Progesterone: WT + anti-Ifnar1, *n* = 16; *Ifnlr1*^−/−^ + anti-Ifnar1, *n* = 11; hSTAT2 KI, *n* = 7; or hSTAT2 KI *Ifnlr1*^*−/−*^, *n* = 5. Estradiol: WT + anti-Ifnar1, *n* = 10; *Ifnlr1*^−/−^ + anti-Ifnar1, *n* = 6. Estradiol and progesterone: WT + anti-Ifnar1, *n* = 9; *Ifnlr1*^−/−^ + anti-Ifnar1, *n* = 13

To assess the effects of IFN-λ signaling on inhibition of ZIKV infection in the FRT during the estradiol phase, we compared intravaginal infection in OVX WT and *Ifnlr1*^−/−^ mice with or without estradiol treatment. We added a blocking anti-Ifnar1 monoclonal antibody (mAb) to OVX, hormone treated, WT, and *Ifnlr1*^−/−^ mice^6,34^ as ZIKV fails to antagonize type I IFN signaling in murine cells^35,36^; indeed, in the absence of anti-Ifnar1 mAb treatment, little infection was observed at 7 dpi in WT or *Ifnlr1*^−/−^ mice (Supplementary Figure 2a–g). Estradiol-treated animals (estrous stage), which showed a thickened epithelial layer (Supplementary Figure 3), were resistant to intravaginal ZIKV infection in anti-Ifnar1 mAb-treated *Ifnlr1*^−/−^ and WT mice (Fig. 3a, c, d). In comparison, mice treated with vehicle, progesterone only (diestrous stage), or estradiol and progesterone (proestrous stage) were infected productively by ZIKV, with RNA levels in all tested fluids and tissues at 7 dpi. To confirm the functional effects of each hormone treatment, we performed whole-genome RNA sequencing analysis on uninfected, OVX, hormone-treated WT mice. Each hormone treatment produced a unique transcriptional profile (Supplementary Figure 4a) with induction of expected, canonical hormone-regulated genes (progesterone: *Gpx3*, *Tcf23*, *Angptl7*, *Ctla2a*, and *Errfi1*; estradiol: *Krt6b*, *Epn3*, *Ablim3*, *Greb1*, and *Rps2*) (Supplementary Figure 4b). These data suggest that type I and type III IFNs do not contribute substantively to the protective effect of estradiol against intravaginal ZIKV infection.

### IFN-λ protection of the vagina is hormone stage-dependent

**Fig. 4:**
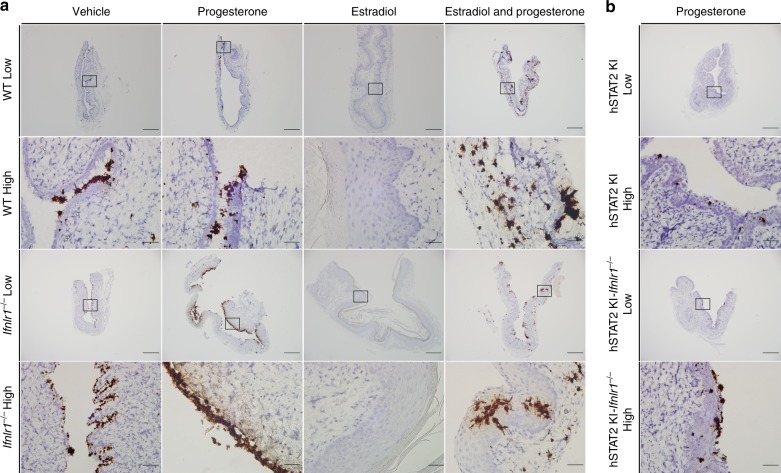
Increased ZIKV infection in the vagina of progesterone-treated OVX *Ifnlr1*^*−/−*^ and hSTAT2 KI *Ifnlr1*^*−/−*^ mice. Six week-old WT, *Ifnlr1*^−/−^, hSTAT2 KI, or hSTAT2 KI *Ifnlr1*^*−/−*^ OVX mice were given a hormone replacement regimen of estradiol, progesterone, estradiol and progesterone, or vehicle. WT and *Ifnlr1*^*−/−*^ mice were treated with 1 mg of anti-Ifnar1 mAb at day −1. On day 0, mice were inoculated via intravaginal route with 10^6^ FFU of ZIKV (Dakar 41525). At 7 dpi, ZIKV RNA was stained using ISH on vaginal tissue (WT and *Ifnlr1*^−/−^, **a**; hSTAT2KI and hSTAT2 KI *Ifnlr1*^−/−^, **b**). Images are representative of two or three experiments. Low magnification scale bar = 500 μm, high magnification scale bar = 50 μm. Numbers of mice analyzed: Vehicle: WT + anti-Ifnar1, *n* = 10; *Ifnlr1*^−/−^ + anti-Ifnar1, *n* = 7. Progesterone: WT + anti-Ifnar1, *n* = 16; *Ifnlr1*^−/−^ + anti-Ifnar1, *n* = 11; hSTAT2 KI, *n* = 7; or hSTAT2 KI *Ifnlr1*^*−/−*^, *n* = 5. Estradiol: WT + anti-Ifnar1, *n* = 10; *Ifnlr1*^−/−^ + anti-Ifnar1, *n* = 6. Estradiol and progesterone: WT + anti-Ifnar1, *n* = 9; *Ifnlr1*^−/−^ + anti-Ifnar1, *n* = 13

Although the vagina is a candidate epithelial barrier site for IFN-λ responses^29^, a recent study showed minimal induction of IFN-α, IFN-β, or IFN-λ during ZIKV or lymphocytic choriomeningitis virus (LCMV) infection^27^. To further evaluate the significance of the IFN-λ response to infection within the FRT, we compared intravaginal infection in WT and *Ifnlr1*^−/−^ mice under hormone conditions that are permissive for ZIKV replication. We again added a blocking anti-Ifnar1 mAb to OVX WT and *Ifnlr1*^−/−^ mice to facilitate infection. Notably, progesterone only-treated OVX *Ifnlr1*^−/−^ mice sustained increased ZIKV infection in vaginal lavage fluid (147-fold, *P* < 0.01) at 3 dpi and in vaginal tissues at 7 dpi (3.3-fold, *P* < 0.01) compared to similarly treated OVX WT mice (Fig. 3a, b). In comparison, the cervix, uterus, serum, brain, and spleen of progesterone only-treated OVX WT and *Ifnlr1*^−/−^ mice had similar levels of viral RNA (Fig. 3c, d Supplementary Figure 5a–c). ZIKV appeared concentrated in the epithelial layer of the vagina in progesterone only-treated mice (Fig. 4a, top left), with *Ifnlr1*^−/−^ mice showing increased intensity of staining (Fig. 4a, bottom left). In comparison, vehicle-treated OVX *Ifnlr1*^−/−^ and WT mice had similar levels of RNA in all tissues tested at 7 dpi. We recently generated an immunocompetent model of ZIKV infection by introducing human *STAT2* into the mouse Stat2 locus (hSTAT2 KI), which allows ZIKV to antagonize innate immune responses in mice. To confirm the hormone dependent effects on IFN-λ activity, we compared ZIKV infection in progesterone only-treated hSTAT2 KI and hSTAT2 KI *Ifnlr1*^−/−^ mice. We observed statistically significant increases in ZIKV infection in the vagina and uterus and a trend toward greater infection in the cervix at 7 dpi of progesterone only-treated OVX hSTAT2 KI *Ifnlr1*^−/−^ compared to hSTAT2 KI mice (Fig. 3e–g). The increased ZIKV infection in vagina of hSTAT2 KI *Ifnlr1*^−/−^ mice was localized to the epithelial layer (Fig. 4b, bottom right).

In contrast to that observed in the diestrous phase after progesterone treatment, we did not detect differences in ZIKV infection of the vagina, cervix, uterus, serum, spleen, or brain in estradiol and progesterone-treated (proestrous phase) OVX WT and *Ifnlr1*^−/−^ mice at 7 dpi in the absence of type I IFN signaling (Fig. 3a–d). RNA ISH also showed relatively equivalent levels of ZIKV infection in the vagina of WT and *Ifnlr1*^−/−^ mice, without substantive differences in inflammation (Fig. 4a, Supplementary Figure 3). These data suggest a hormone stage-dependent antiviral effect of IFN-λ in the context of ZIKV infection of the vagina.

**Fig. 5:**
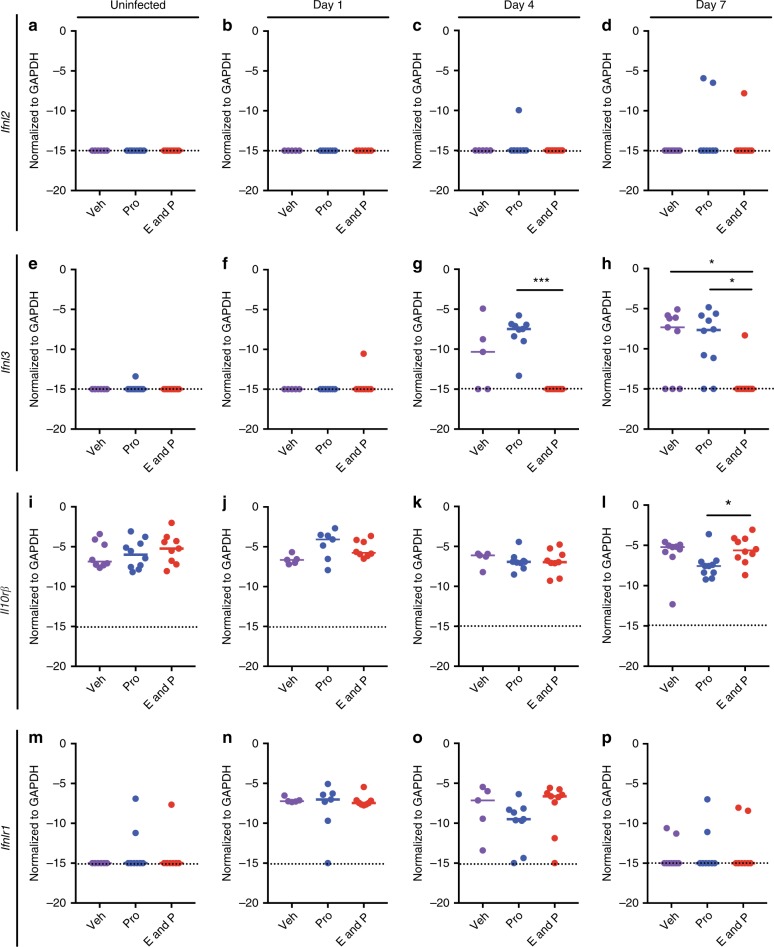
IFN-λ and its receptor expression are hormone-dependent after intravaginal ZIKV infection. Six-week-old WT OVX mice were given hormone replacement regimens of vehicle, progesterone-only, or a combination of estradiol and progesterone. Mice were treated with 1 mg of anti-Ifnar1 mAb at day −1. On day 0, mice were inoculated via intravaginal route with 10^6^ FFU of ZIKV (Dakar 41525); 1, 4, or 7 days after infection, total RNA was extracted, and mRNA expression analyzed by RT-qPCR (*Ifnl2* **a**–**d**; *Ifnl3* **e**–**h**; *Il10r*β **i**–**l**; *Ifnlr1* **m**–**p**). Uninfected animals (for comparison) were harvested at the day 7 time point. Dotted lines indicate the LOD. Results are pooled from two or three experiments. Bars indicate median values (Kruskal–Wallis test: *, *P* < 0.05, ***, *P* < 0.001). Numbers of mice: Uninfected vehicle *n* = 8; Uninfected progesterone n = 10; uninfected estradiol and progesterone n = 9; day 1, vehicle n = 5; day 1, progesterone *n* = 7; day 1, estradiol and progesterone *n* = 8; day 4, vehicle *n* = 5; day 4, progesterone *n* = 9; day 4, estradiol and progesterone *n* = 9; day 7, vehicle *n* = 9; day 7, progesterone *n* = 10; and day 7, estradiol and progesterone *n* = 10

The disparity of ZIKV infection in *Ifnlr1*^−/−^ mice after different hormone treatments might be due to differential expression of IFN-λ or its heterodimeric receptor Ifnlr1/Il10rβ. To evaluate this hypothesis, we measured the levels of *Ifnlr1*, *Il10rb*, *Ifnl2*, and *Ifnl3* mRNA by RT-qPCR in the vagina on days 1, 4, and 7 after ZIKV infection (Fig. 5). *Ifnl1* is a pseudogene and the genomic region of *Ifnl4* is missing in mice^37,38^ and were not measured. Consistent with our *Ifnlr1*^−/−^ mouse data, estradiol and progesterone-treated mice had little or no *Ifnl2* and *Ifnl3* induced 1, 4 or 7 dpi (Fig. 5a–h). However, *Ifnl3* mRNA was detected in vehicle and progesterone only-treated animals (Fig. 5g, h). This suggests that when estradiol is absent, the vagina induces IFN-λ in response to ZIKV infection to reduce viral burden in the vaginal epithelium. In contrast, in the estradiol and progesterone proestrous stage, IFN-λ is not induced sufficiently to confer an antiviral effect in the vagina. Of note, only small changes in expression of *Il10rb* were observed between days 0 and 7 (Fig. 5i–l), whereas *Ifnlr1* mRNA was time-dependent, with the highest expression occurring on days 1 and 4 after infection (Fig. 5m–p).

**Fig. 6:**
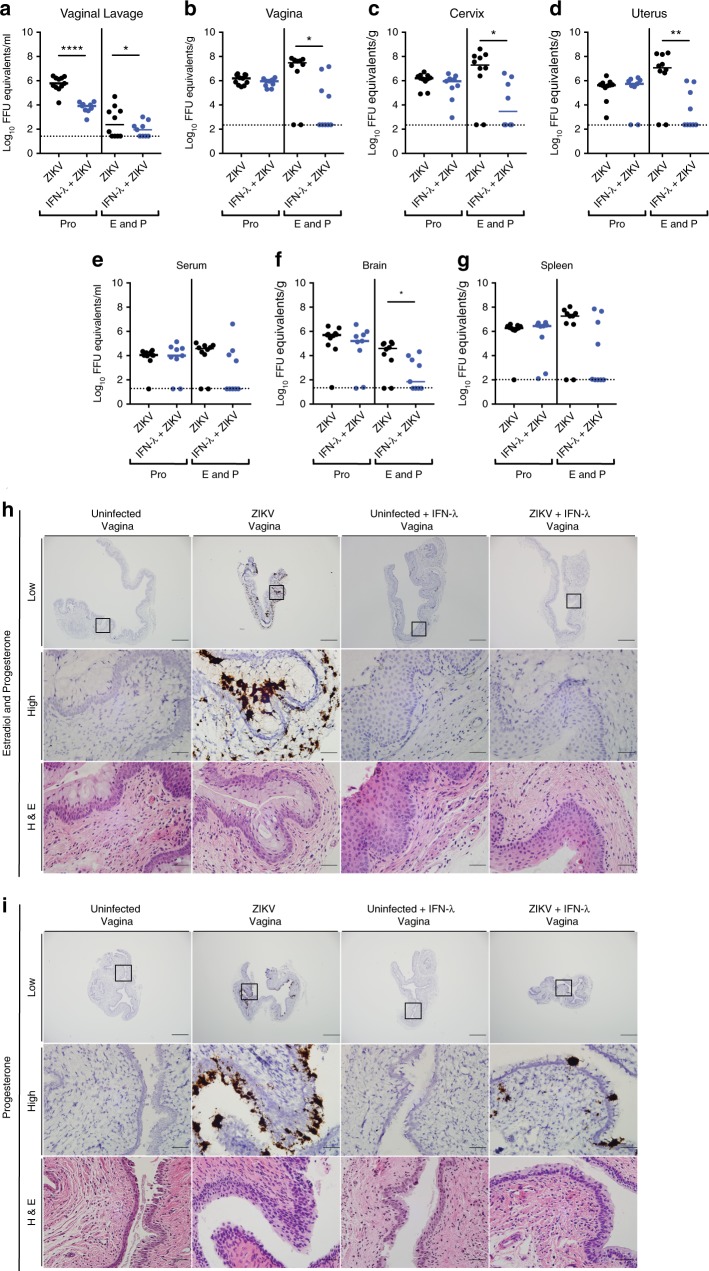
Pegylated IFN-λ2 protects against intravaginal ZIKV infection in estradiol and progesterone-treated OVX WT mice. Six-week-old WT OVX mice were given a hormone replacement regimen of progesterone or estradiol and progesterone. Mice were treated with 1 mg of anti-Ifnar1 mAb at day −1. On day 0, mice were treated via intravaginal route with 25 μg of pegylated IFN-λ2. Eight hours later, mice were inoculated with 10^6^ FFU of ZIKV (Dakar 41525). At 3 dpi, a vaginal lavage was performed (**a**). At 7 dpi, ZIKV RNA was measured from the vagina (**b**), cervix (**c**), uterus (**d**), serum (**e**), brain (**f**), and spleen (**g**). Dotted lines indicate the LOD. Results are pooled from two or three experiments. Bars indicate median values (Mann–Whitney test: *, *P* < 0.05, **, *P* < 0.01, ****, *P* < 0.0001). ISH and H&E staining were performed on tissues from the vagina (Estradiol and progesterone-treated mice, **h**; Progesterone-treated mice, **i**). Numbers of mice: Progesterone: WT + anti-Ifnar1 mAb, IFN-λ2, *n* = 9; WT + anti-Ifnar1 mAb, *n* = 11. Estradiol and progesterone: WT + anti-Ifnar1 mAb, IFN-λ2, *n* = 9; WT + anti-Ifnar1 mAb, *n* = 10. Representative images are shown

Although OVX WT and *Ifnlr1*^−/−^ mice given both estradiol and progesterone had equivalent ZIKV infection in FRT tissues, we speculated that cells in the vagina might respond to exogenous IFN-λ. To test this hypothesis, OVX WT mice treated with estradiol and progesterone and anti-Ifnar1 mAb were given pegylated mouse IFN-λ2 via an intravaginal route 8 h before local inoculation with ZIKV (Fig. 6). IFN-λ-treated mice sustained lower (1274- to 137,019-fold, *P* < 0.05) infection in the vaginal lavage fluid, vagina, cervix, and uterus (Fig. 6a–d), and this effect prevented dissemination to the serum, spleen, and brain (Fig. 6e–g). The decreased levels of ZIKV infection after pegylated IFN-λ2 treatment was confirmed by RNA ISH, with markedly less viral RNA observed in the vagina and the muscle layer of the uterus (Fig. 6h, Supplementary Figure 6). To further explore the possible utility of IFN-λ for preventing sexual transmission of ZIKV, we administered pegylated IFN-λ2 to progesterone-only treated animals. Although we observed a significant decrease in viral RNA levels in vaginal lavage fluid at 3 dpi (73-fold, *P* < 0.0001) (Fig. 6a), similar levels were present in the remainder of the FRT and peripheral tissues at 7 dpi (Fig. 6b–g). RNA ISH on vaginal tissues also showed equivalent levels of ZIKV RNA in the epithelial layer (Fig. 6i). Thus, while IFN-λ signaling does not have an endogenous antiviral function in the vagina in estradiol and progesterone-treated mice, exogenous administration can prevent local infection and spread of ZIKV. In contrast, in progesterone only-treated mice, exogenous administration of IFN-λ had less antiviral effect even though higher levels of ZIKV RNA were observed in the vagina of anti-Ifnar1 mAb-treated *Ifnlr1*^−/−^ or hSTAT2 KI *Ifnlr1*^−/−^ mice (see Fig. 3a, b, e). These phenotypes suggest that during the diestrous phase, IFN-λ produced in the context of infection has a saturating antiviral effect in the vagina.

## Discussion

Prior studies have established that ZIKV infects specific cell types at the maternal–fetal interface, in the brain and eye, and in the male reproductive tract^1,6,39–41^. Our experiments in primary human cells and mice examined the antiviral role of type I and III IFNs on intravaginal ZIKV infection. IFN-λ and IFN-β induced an ISG signature in primary human vaginal and cervical epithelial cells that contributed to protection against ZIKV infection. We confirmed that the estrous phase of the reproductive cycle was protective against intravaginal infection^17,18^, and using transgenic mice established that this process was independent of type I and III IFNs. Because IFN-λ is a cytokine that acts primarily at barrier surfaces^29^, we evaluated its impact on ZIKV infection in the vagina. Unexpectedly, the inhibitory effect of IFN-λ in the vagina was hormone stage dependent in mice. Although estradiol and progesterone-treated mice produced low levels of IFN-λ after intravaginal ZIKV inoculation, intravaginal administration of pegylated IFN-λ2 protected the FRT from ZIKV infection and subsequent dissemination. Reciprocally, progesterone only-treated animals induced IFN-λ3 after ZIKV infection but unexpectedly did not show an antiviral effect of exogenous, local pegylated IFN-λ2 treatment.

The estrous cycle and female sex hormones can modulate the susceptibility to sexually transmitted infections including HSV, human papilloma virus, human immunodeficiency virus, *Neisseria gonorrhoeae*, and *Chlamydia trachomatis*^21,23^. In the estradiol-high estrous phase, the vagina is more resistant to infection although the basis for this phenotype is incompletely understood. In comparison, in progesterone-high diestrous phase, when the reproductive tract prepares for implantation, the epithelial barrier of the vagina is more susceptible to sexually transmitted infections^21^. Consistent with these concepts, we show that OVX mice were susceptible to ZIKV through the intravaginal route when administered progesterone with or without estradiol; in comparison, mice given only estradiol were resistant to ZIKV infection. Since vehicle-treated OVX mice were susceptible to ZIKV as well, we confirmed that estradiol alone had an active role in suppressing virus infection. These data agree with a prior report showing that mice in the diestrous, but not estrous, phase were susceptible to intravaginal infection of ZIKV^18^. Although progesterone and estradiol differentially condition the vaginal epithelium and underlying tissue, the mechanisms for the disparate susceptibility to sexually transmitted infections remain unclear^21^. Although one study in mice suggested an estrous cycle-dependent protective role of IFN-ε against infections in the vagina^26^, in our model, estradiol treatment conferred protection against intravaginal ZIKV infection in mice with acquired or genetic deficiencies in type I IFN signaling, suggesting that IFN-ε was not the dominant antiviral cytokine during the estrous phase. Since estradiol promotes proliferation of the vaginal epithelium, a plausible explanation for the reduced susceptibility to sexually transmitted infections during the estrous phase is that the thickened, keratinized barrier is inherently less permeable to pathogens^33,42,43^.

A recent study suggested that sexual transmission of ZIKV may be underappreciated and contribute more significantly to sustaining the virus in human population^12^. Because ZIKV can disseminate to the placenta and fetus after intravaginal infections^11,17,44,45^, sexual transmission might account for greater transmission and number of congenital infections. Thus, there is an urgent need to develop countermeasures that protect against this transmission route. Epithelial and mucosal surfaces can respond to IFN-λ, and IFN-λ is induced after viral (e.g., reovirus, rotavirus, norovirus, and influenza A virus) infection to higher levels then type I IFN at these sites^46–49^. Also, IFN- λ blocks HSV-2 replication in the vaginal mucosa, in contrast to IFN-α, which does not^50^. We examined the effect of endogenous IFN-λ signaling in the vagina because of its recently described activity against ZIKV in the placenta^30,32^. When mice were treated with progesterone-only, IFN-λ3 was induced, and *Ifnlr1*^*−/−*^ mice had greater levels of ZIKV RNA in the vaginal epithelium than WT mice. However, IFN-λ2 was not induced in the vagina, which could be due to sequence differences in the promoter region of the genes. In HIV-infected macrophages, IFN-λ1 and IFN-λ3 induced more ISGs than IFN-λ2 and conferred better protection^51^. Our results ostensibly contrast with data from medroxyprogesterone-treated, non-OVX mice, which suggested only limited IFN-λ signaling after ZIKV infection^27^. In that study, IFN-λ signaling was upregulated in the vagina after treatment with acitretin, a retinoic acid derivative, or prior infection with LCMV^27^. The differences in IFN-λ induction and signaling between the two studies could reflect the experimental design, relative levels of progesterone administered, or disparate use of anti-Ifnar1 mAb. Apart from this, when exogenous, pegylated IFN-λ2 was administered via intravaginal route immediately before ZIKV infection to mice treated with only progesterone, no additional protection was observed. These data contrast with an IFN-λ treatment experiment in medroxyprogesterone-treated animals where reductions in HSV-2 titers were seen in vaginal washes^52^. However, in that study, no other tissue titers (vagina, cervix, uterus) were analyzed and no other hormonal conditions (e.g., estrogen and progesterone) were tested. Although further studies are warranted, we speculate that the amount of endogenous IFN-λ produced in the progesterone-only mice provides a saturating antiviral signal such that no further benefit is acquired from exogenous IFN-λ treatment. Alternatively, factors in the mucosal environment (e.g., microbiome, pH, mucus, or polarity of Ifnlr1/IL10rβ receptors on epithelium) under progesterone-only conditions could impact the signaling potential of exogenous IFN-λ.

To simulate the stages in the estrous cycle when both progesterone and estradiol levels are produced, we treated OVX mice with a combination of both hormones. We observed no differences in ZIKV infection in the FRT of *Ifnlr1*^−/−^ and WT mice treated with both estradiol and progesterone. These results are consistent with our data showing that ZIKV infection in the vaginas of estradiol and progesterone treated mice did not induce IFN-λ. However, when estradiol and progesterone-treated mice were given with pegylated IFN-λ2, most animals were completely protected against ZIKV infection. Studies in mice and possibly NHPs that further define the hormonal stage dependence, the durability of effect, and local inflammatory responses are needed to determine the feasibility of IFN-λ as a countermeasure against sexual transmission of ZIKV. In support of its possible utility, primary human vaginal and cervical epithelial cells responded to IFN-λ treatment with antiviral responses that blocked ZIKV infection. One potential advantage of IFN-λ over IFN-α/β intravaginal administration is that it is less inflammatory in nature^29,53,54^, which could enhance tolerability.

The establishment of mouse models of ZIKV infection in the lower FRT after intravaginal inoculation may be useful for testing therapeutic agents or vaccines. Our data in mice and primary human cells suggest that IFN-λ is a key cytokine at the epithelial layer of the vagina and its induction and subsequent action depends on the hormone-dependent stage of the estrous cycle. Countermeasures against ZIKV and likely other sexually transmitted infections may need to account for differences in hormonal status and innate immune defense programs that occur in non-pregnant and pregnant women.

## Methods

### Ethics statement

This study was carried out in accordance with the recommendations in the Guide for the Care and Use of Laboratory Animals of the National Institutes of Health. The infection and surgery protocols were approved by the Institutional Animal Care and Use Committee at the Washington University School of Medicine (Assurance number A3381-01, Protocols 20150222 and 20150034). Mice were anaesthetized with a ketamine hydrochloride and xylazine mixture or isoflurane when inoculations or procedures were performed to reduce animal discomfort and suffering. Animals received sustained-released buprenorphine and carprofen for pain management.

### Viruses

ZIKV strain Dakar 41525 (Senegal, 1984) was provided by the World Reference Center for Emerging Viruses and Arboviruses (R. Tesh). A mouse-adapted variant of Dakar 41525 was used and has been described^55^. ZIKV strain Paraiba (Brazil, 2015) was provided by S. Whitehead (NIH, Bethesda, MD)^56^. All virus stocks were propagated in mycoplasma-free Vero cells (American Tissue Culture Collection) and titrated by focus-forming assay (FFA), as described previously^6^.

### Mouse studies

Six-week-old, WT C57BL/6J female mice were purchased from Jackson Laboratories (# 000664). *Ifnlr1*^−/−^^50^ and *Ifnar1*^−/−^ mice were backcrossed onto a C57BL/6J background. STAT2 KI mice on a C57BL/6J background were recently described^55^. hSTAT2 KI *Ifnlr1*^*−/−*^ mice were generated by standard F2 crossing. All mice were bred in a pathogen-free facility at the Washington University School of Medicine.

OVX surgeries were performed on 6-week-old WT, *Ifnar1*^−/−^, *Ifnlr1*^−/−^, hSTAT2 KI, or hSTAT2 KI *Ifnlr1*^*−/−*^ mice. Under anesthesia, a small dorsal midline incision was made below the sternum, and the skin was separated from the underlying abdominal wall using a scissors. A small incision was made in the abdominal cavity and using forceps, the ovary was removed from the peritoneal cavity and cauterized below the oviduct. The remaining tissue was placed back into the peritoneal cavity, and the incision area was sutured closed. The process was repeated on the contralateral side. After a 1-week recovery period, hormone pellets (Innovative Research of America) were implanted or soluble hormones (Sigma-Aldrich) were suspended in sunflower oil and injected. Time-released pellets were placed under the skin, releasing 10 mg of progesterone (P4) or 0.1 mg of 17β-estradiol, or vehicle pellets (that contained no hormones) over 21 days for an average dose of 500 μg or progesterone and/or 5 μg of estradiol per day. For hormone injections, mice were administered oil only (vehicle), 200 μg of estradiol and/or 2 mg of progesterone subcutaneously in the back of the animals. After hormone treatment, mice were treated with 1 mg of anti-Ifnar1 mAb (MAR1-5A3, produced by Leinco Technologies)^57^ and inoculated intravaginally with 10^6^ FFU of ZIKV (Dakar 41525). At 3 dpi, a vaginal lavage was performed: two 30 μL washes with PBS were added to 30 μL of DMEM supplemented with 5% FBS and frozen at −80 °C. Tissues from WT and *Ifnlr1*^*−/−*^, hSTAT2 KI, or hSTAT2 KI *Ifnlr1*^*−/−*^ mice were harvested at 7 dpi. Tissues from *Ifnar1*^*−/−*^ mice were harvested at 6 dpi. No differences in the different hormone treatments (pellets versus injections) were observed in terms of estrous cycle or susceptibility to ZIKV infection.

### IFN-λ treatment of mice

OVX WT C57BL/6 J were treated with estradiol and progesterone or progesterone-only as described above. One week later, mice received 1 mg of anti-Ifnar1 mAb, and the following day some animals received 25 μg of pegylated IFN-λ2 (Bristol-Myers Squibb) via intravaginal route. Eight hours later, mice were inoculated via intravaginal route with 10^6^ FFU of ZIKV (Dakar 41525) and processed at 3 and 7 dpi as described above.

### Human vaginal and cervical epithelial cell experiments

HVECs and HCECs from four or three deceased healthy donors, respectively (purchased from Lifeline Cell Technology), were maintained in ReproLife Reproductive Medium (Lifeline) according to manufacturer’s instructions. Information on the menstrual phase of human donors was not available, as the samples were de-identified. Cells were pretreated with human recombinant IFN-β or IFN-λ (100 ng/mL; R&D Systems) for ~18 h in quadruplicate before harvest, total RNA was extracted using a GenElute Total RNA Miniprep Kit (Sigma). Following isolation and treatment with DNAse (Sigma), RNA was reverse transcribed using an iScript cDNA synthesis kit (BioRad) containing 1 μg of total RNA. ISG mRNA expression was determined by RT-qPCR using IQ SYBR Green Supermix (BioRad) in a BioRad CFX96 touch real time PCR detection system or an Applied Biosystems StepOnePlus real time PCR machine as reported previously^32,58^. Primer sequences are GAPDH (5′-GAAGGTCGGAGTCAACGGATTT-3′ and 5′-GAATTTGCCATGGGTGGAAT-3′), IFI44L (5′-TGCAGAGAGGATGAGAATATC-3′ and 5′- ACTAAAGTGGATGATTGCAG-3′), OAS1 (5′-ATAAAAGCAAACAGGTCTGG-3′ and 5′- TCTGGCAAGAGATAGTCTTC-3′), OASL (5′-GTACCAGCAGTATGTGAAAG-3′ and 5′-ATGGTTAGAAGTTCAAGAGC-3′), and MX1 (5′-F: CTGACTCTAATAGACCTTCCTG-3′ and R: 5′-GATCTTATACCCAATGTCAGC-3′). Cells were inoculated with ZIKV (Paraiba, MOI of 3) for 48 h after pretreatment with medium, IFN-β or IFN-λ. Viral burden was determined from supernatants using an FFA as previously described^31^.

### Virus titration

Tissues were homogenized in 300–600 μL of DMEM supplemented with 10% FBS using stainless-steel beads in a Bullet Blender Storm 24 homogenizer instrument (Next Advance) for 1–2 min. Homogenates were clarified by centrifugation at 6000×*g* for 5 min and stored at −80 °C. RNA was extracted using an Applied Biosystems 5x MagMax RNA 96 viral isolation kit (Thermo Scientific) and a Kingfisher duo prime extraction machine (Thermo Scientific). ZIKV RNA levels were determined by one-step quantitative RT–qPCR using an Applied Biosystems Taqman RNA-to-Ct 1-step kit (Thermo Scientific) on an ABI 7500 Fast Instrument using standard cycling conditions. Published primer/probe sets for RT-qPCR of ZIKV were used^1^.

1183F: 5′-CCACCAATGTTCTCTTGCAGACATATTG-3′;

1268R: 5′-TTCGGACAGCCGTTGTCCAACACAAG-3′;

and probes (1213F): 5′-56-FAM/AGCCTACCT/ZEN/TGACAAGCAGTC/3IABkFQ-3′.

Viral burden was expressed on a log_10_ scale as ZIKV RNA equivalents per gram or milliliter after comparison with a standard curve produced using serial tenfold dilutions of ZIKV RNA^1^.

### Histology and RNA ISH

Tissues were collected and fixed overnight in 4% paraformaldehyde (PFA) in PBS. Vaginal and uterine tissues from uninfected and infected mice were cut into 3-μm-thick sections followed by hematoxylin and eosin (H&E) staining. RNA ISH was performed using RNAscope 2.5 (Advanced Cell Diagnostics) according to the manufacturer’s instructions. PFA-fixed paraffin-embedded tissue sections were deparaffinized by incubating for 60 min at 60 °C. Endogenous peroxidases were quenched with H_2_O_2_ for 10 min at room temperature. Slides were boiled for 15 min in RNAscope Target Retrieval Reagents and incubated for 30 min in RNAscope Protease and before probe hybridization. The probe targeting ZIKV RNA was designed and synthesized by Advanced Cell Diagnostics (Catalog #467871). Positive (targeting *plr2a* gene) and negative (targeting bacterial gene *dapB*) control probes also were obtained from Advanced Cell Diagnostics (Catalog #312471 and #310043, respectively) and used for all tissue staining. Slides were counterstained with Gill’s hematoxylin and visualized using bright-field microscopy.

### IFN-λ RT-qPCR in mouse vaginal tissue

Vaginal tissues were harvested at 1, 4, and 7 dpi from hormone or vehicle-treated OVX mice and homogenized as described above. Total RNA was extracted using a Quick-RNA Microprep Kit (Zymo Research Catalog #11-327M) and DNAse treatment was performed. *Ifnlr1, Il10r*β*, Ifnl2*, and *Ifnl3* were quantified by RT-qPCR as described above and normalized to *Gapdh* expression. The following PrimeTime primers (IDT) were used: *Ifnlr1*: Mm.PT.58.10781457, *Il10r*β: Mm.PT.58.29421186, *Ifnl2*: Mm.PT.58.31485549, and *Ifnl3*: Mm.PT.58.8956530, and *Gapdh*: Mm.PT.39a.1.

### RNAseq on IFN-treated HVECs

Total RNA was extracted as described above, and RNASeq was performed as described^31,32,58^. Briefly, libraries were prepared with the Ultra Library Preparation kit (New England BioLabs), and quality was determined using the Qubit assay (Thermo Scientific) and Agilent 2100 Bioanalyzer. Sequencing was performed with the Illumina HiSeq2500 rapid-run mode, and CLC Genomics Workbench 9.0 (Qiagen) was used to map sequence data to the human reference genome (hg19). Differentially expressed genes were identified using the DESeq2 package in R^59^ with a significance cut-off described in the Figure legends. Heatmaps were generated using MeV software and were based upon log(RPKM) values. Gene set enrichment analysis (GSEA) v2.0^60^ was used for enrichment analyses, with false discovery rates (FDRs) described in the figure legends. Files associated with RNASeq from the current study have been deposited into Sequence Read Archive Files (SRA).

### RNAseq on OVX hormone-treated WT mice

OVX and hormone treatment was performed on WT mice as described above. Total RNA was extracted from vaginal tissue 7 days after hormone treatment began as described above. Samples were processed for RNA-seq analysis at the Genome Techonology Access Center at Washington University. Fragments were sequenced on an Illumina HiSeq-2500 using single reads extending 50 bases. Briefly, RNA-seq reads were aligned to the Ensembl 76 top-level assembly with STAR (version 2.0.4b). Gene counts were derived from the number of uniquely aligned unambiguous reads by Subread:featureCount (version 1.4.5). Transcript counts were produced by Sailfish (version 0.6.3). Sequencing performance was assessed for total number of aligned reads, total number of uniquely aligned reads, genes and transcripts detected, ribosomal fraction, junction saturation, and read distribution over known gene models with RSeQC (version 2.3). All gene-level counts were imported into the R/Bioconductor package Deseq2 and normalized values (rlog normalization) were used for heatmap generation using the Phantasus* web-service (https://artyomovlab.wustl.edu/phantasus/).

### Statistical analysis

Unless otherwise specified, all data were analyzed with GraphPad Prism and DeSeq2 package in R software. Statistical significance of Gene Set Enrichment Analysis (GSEA) was based on the family wise-error rate. For viral burden analysis, the log_10_ transformed titers were analyzed by Mann–Whitney, one-way ANOVA, or a Kruskal–Wallis test with multiple comparison correction. A *P* value of <0.05 indicated statistically significant differences.

### Reporting Summary

Further information on experimental design is available in the Nature Research Reporting Summary linked to this article.

## Supplementary information

Supplementary Information

Reporting Summary

Source data

## Data Availability

All data generated or analyzed during this study are included in this published article and its supplementary information files or have been placed in public repositories (RNAseq data, described above). RNAseq data on OVX hormone-treated WT mice have been deposited in GEO (GSE122591). RNAseq on IFN-treated human vaginal epithelial cells data number is deposited in Sequence Read Archives (PRJNA506090). All data and materials are available upon request.
