# Supplementary material for: Interferon lambda protects the female reproductive tract against Zika virus infection
Source: Nat Commun. 2019 Jan 17;10:280. doi: 10.1038/s41467-018-07993-2 (PMC6336786; doi:10.1038/s41467-018-07993-2)
Supplement: Supplementary file 1 — Supplementary Information [file 41467_2018_7993_MOESM1_ESM.pdf]

Interferon lambda protects the female reproductive tract against Zika virus infection

Caine et al.

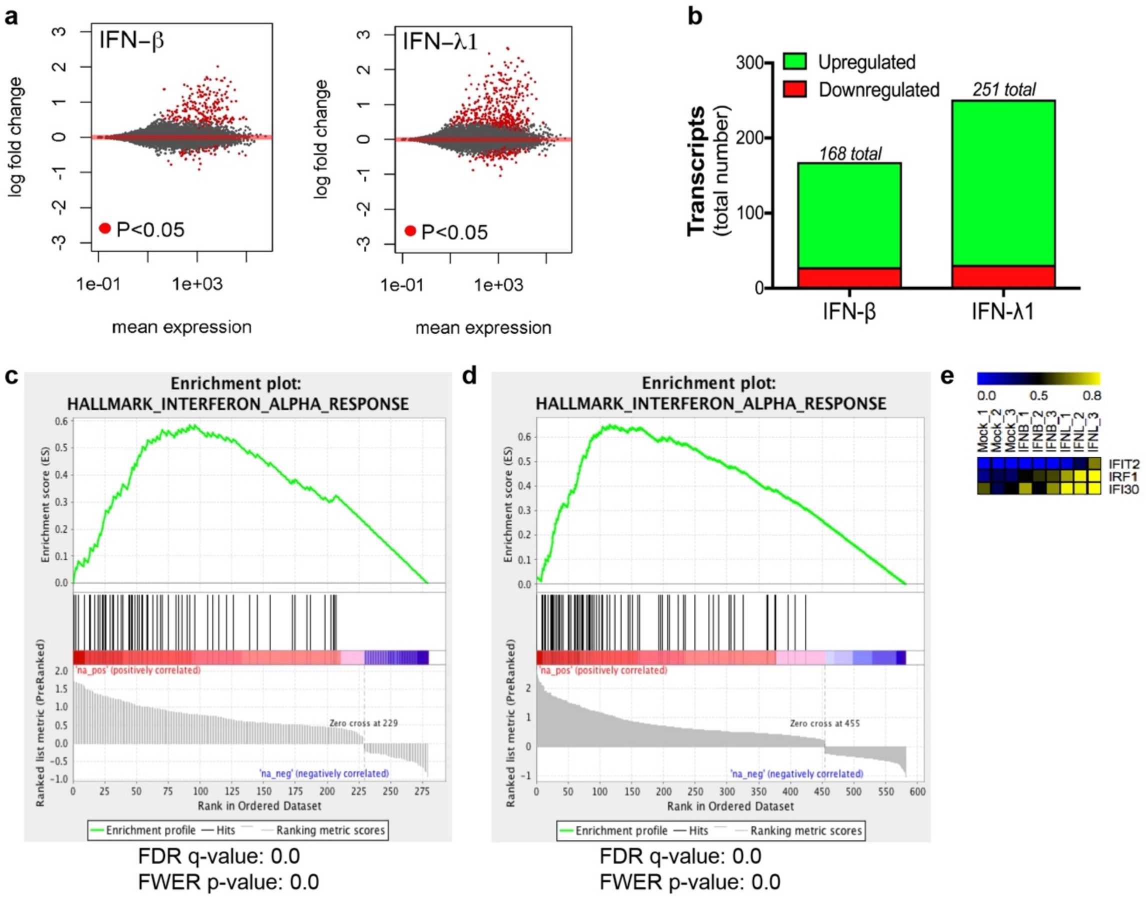

Supplementary Figure 1. IFN-λ and IFN-β upregulates multiple ISGs in primary human vaginal cells. (**a**) MA plots generated in R following DeSeq2 analysis demonstrating the differential expression of transcripts between primary HVECs treated with 100 ng/mL of IFN-β (*left*) or IFN-λ1 (*right*) for ~16 h relative to mock-treated controls. Data are plotted as $\log_2$ fold changes (y-axis) and mean expression (x-axis). Red symbols denote transcripts whose expression was differentially expressed at $P < 0.05$. (**b**) Graph demonstrating the number of transcripts upregulated (in green) or downregulated (in red) following IFN-β or IFN-λ1 treatment of HVECs. (**c, d**) Graphs showing enrichment plots generated by Gene Set Enrichment Analysis (GSEA) for the interferon alpha pathway induced by IFN-β (**c**) or IFN-λ1 (**d**) treated HVECs. *P* values are stated at the bottom and were determined by the family wise-error rate. (**e**) Heat map (based on log(RPKM) values) of selected ISGs that are differentially induced by IFN-λ1 treatment (relative to IFN-β) of HVECs. Color intensity indicates the level of gene expression (yellow for up-regulation and blue for down-regulation).

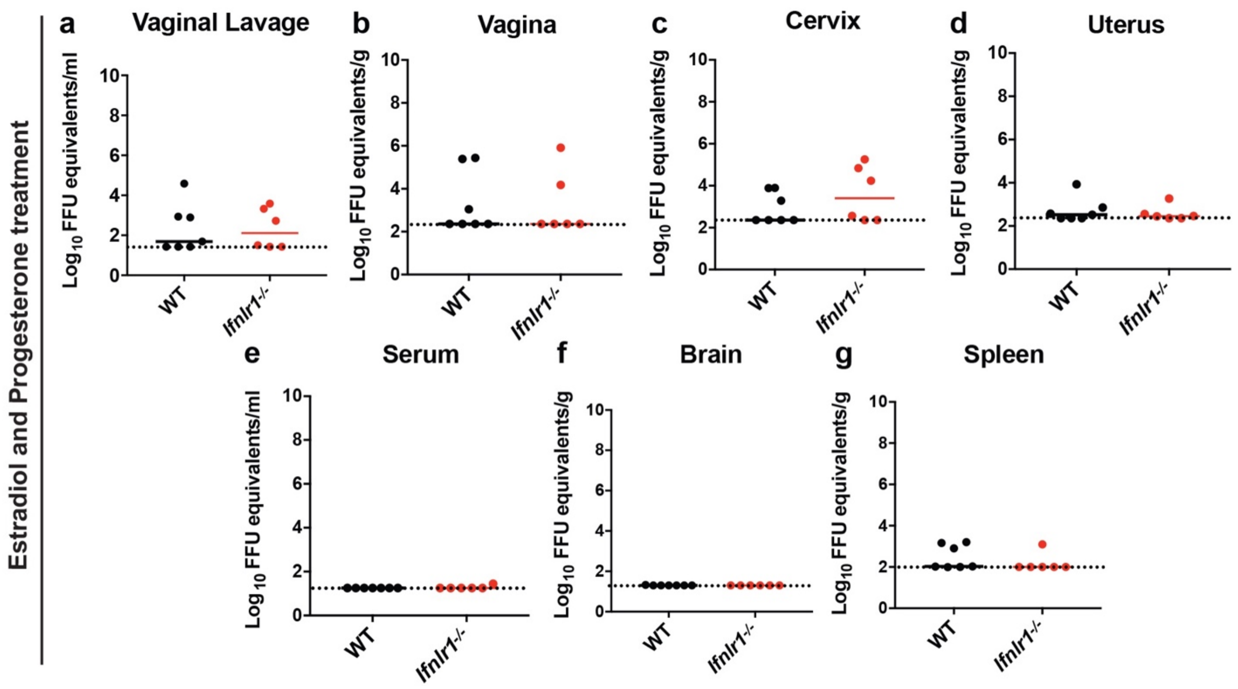

Supplementary Figure 2. Levels of ZIKV RNA in the FRT of WT and *Ifnlr1$^{-/-}$* mice in the absence of anti-Ifnar1 mAb treatment. Six week-old WT or *Ifnlr1$^{-/-}$* OVX mice were given a hormone replacement regimen of estradiol and progesterone. Mice were inoculated via intravaginal route with 10^6 FFU of ZIKV. At 3 dpi, vaginal lavage was performed (**a**). At 7 dpi, ZIKV RNA was measured from the vagina (**b**), cervix (**c**), uterus (**d**), serum (**e**), brain (**f**), and spleen (**g**). Dotted lines indicate the LOD. Results are pooled from two or three experiments. Mann-Whitney test with $P < 0.05$ was performed. Bars indicate median values. Numbers of mice: WT, n = 7; *Ifnlr1$^{-/-}$*, n = 6.

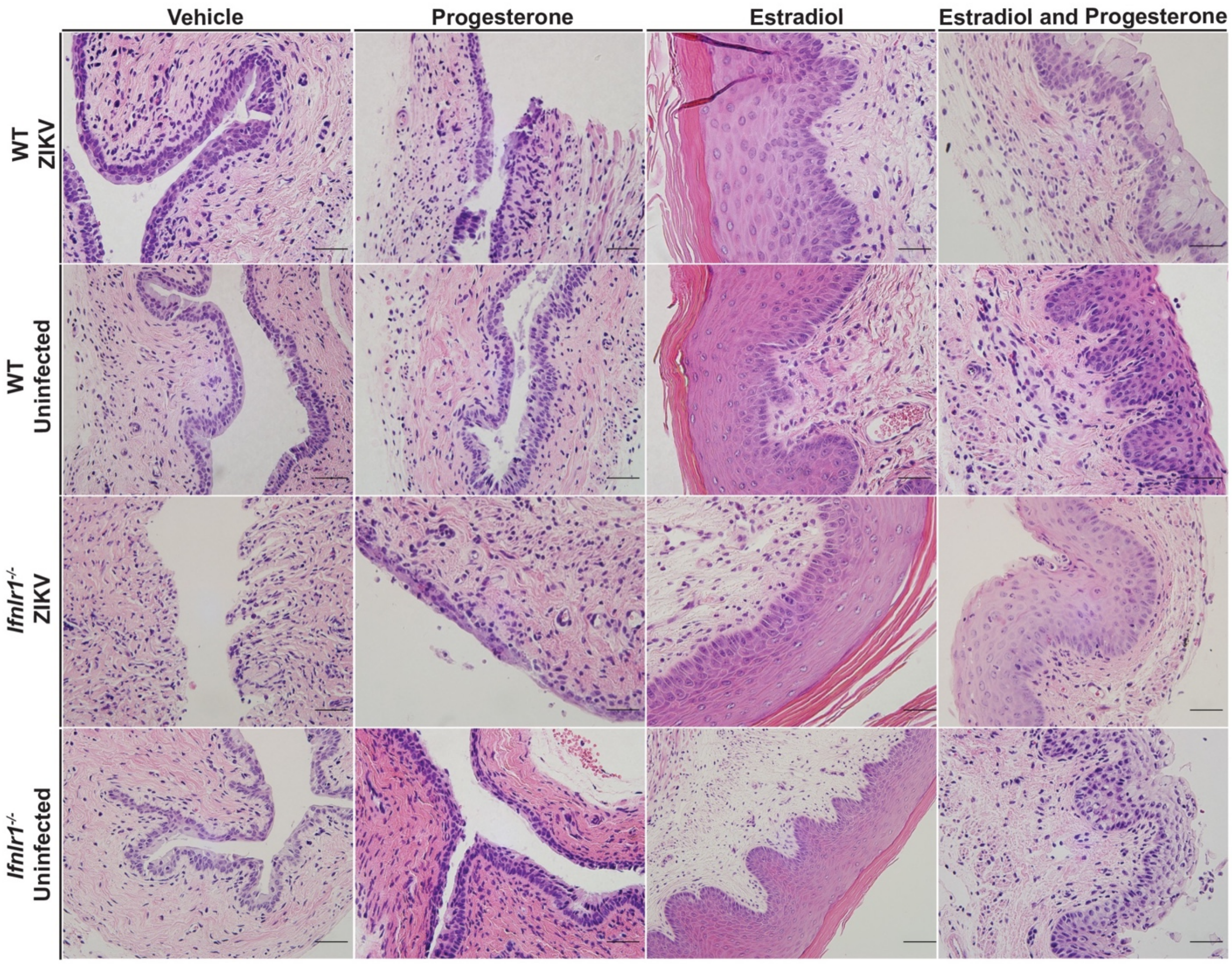

Supplementary Figure 3. Exogenous reproductive hormone treatment modulates thickness of vaginal epithelium but not levels of inflammation in WT and *Ifnlr1*$^{-/-}$ OVX mice. Six week-old WT or *Ifnlr1*$^{-/-}$ OVX mice were given a hormone replacement regimen of estradiol, progesterone, a combination of estradiol and progesterone, or vehicle. Mice were inoculated via intravaginal route with 10^6 FFU of ZIKV (Dakar 41525). One day prior to infection, mice were treated with 1 mg of anti-Ifnar1 mAb. At 7 dpi, vaginal tissues were stained using H & E. Results are pooled from two or three experiments. Low magnification scale bar = 500 µm, high magnification scale bar = 50 µm. Numbers of mice: Vehicle: WT + anti-Ifnar1, n = 10; *Ifnlr-/-* + anti-Ifnar1, n = 7. Progesterone: WT + anti-Ifnar1, n = 16; *Ifnlr1*$^{-/-}$ + anti-Ifnar1, n = 11; Estradiol: WT + anti-Ifnar1, n = 10; *Ifnlr1*$^{-/-}$ + anti-Ifnar1, n = 6. Estradiol and Progesterone: WT + anti-Ifnar1, n = 9; *Ifnlr1*$^{-/-}$ + anti-Ifnar1, n = 13. Representative images are shown.

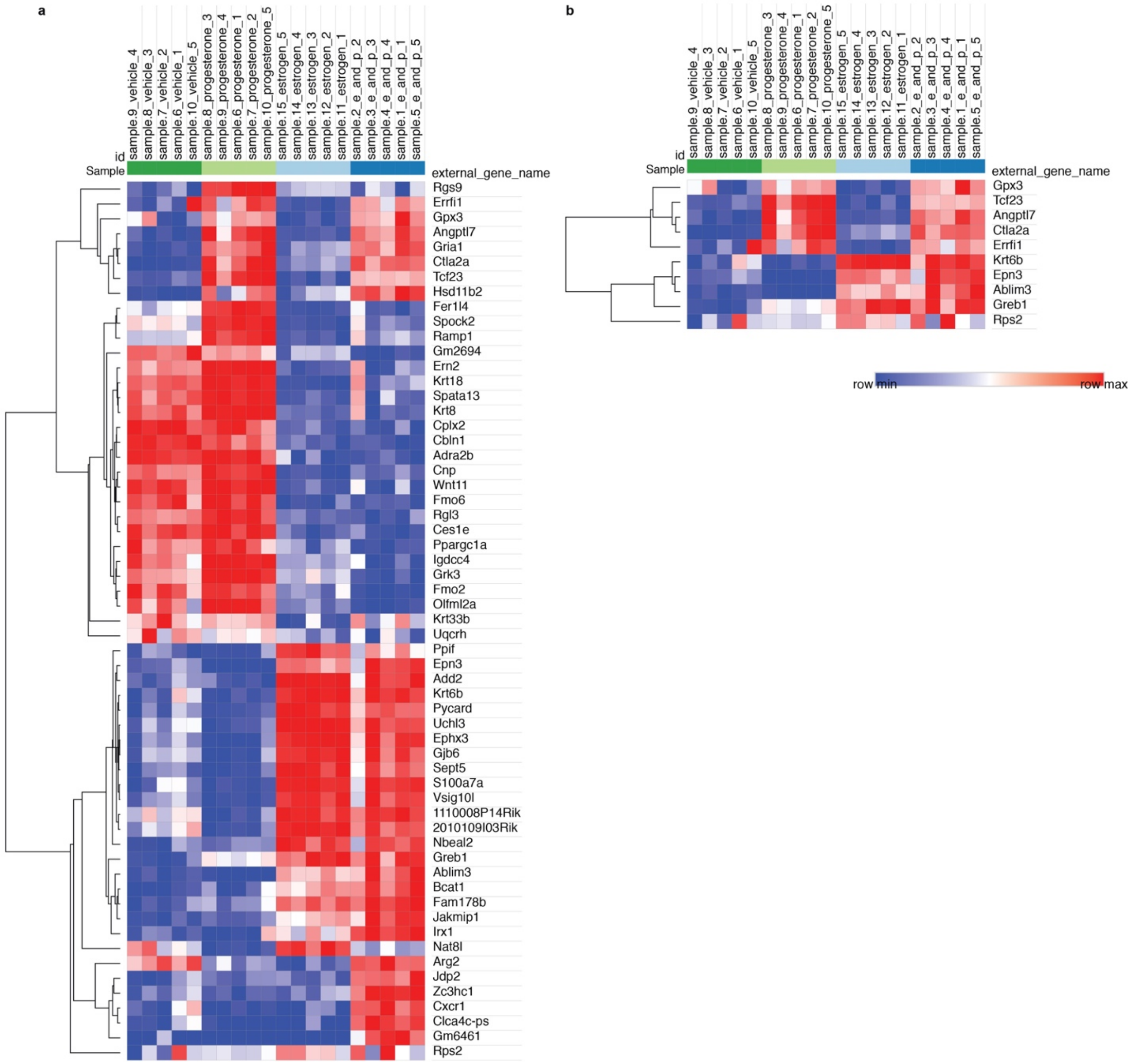

Supplementary Figure 4. Exogenous reproductive hormone treatment produced unique transcriptional profiles in WT OXV mice. Six week-old WT OVX mice were given a hormone replacement regimen of estradiol, progesterone, a combination of estradiol and progesterone, or vehicle. One week following the beginning of hormone treatment, total RNA was extracted from total vaginal tissue. RNASeq analysis showed unique transcriptional profiles for each hormone treatment (**a**). Canonical hormone-regulated genes were induced (**b**).

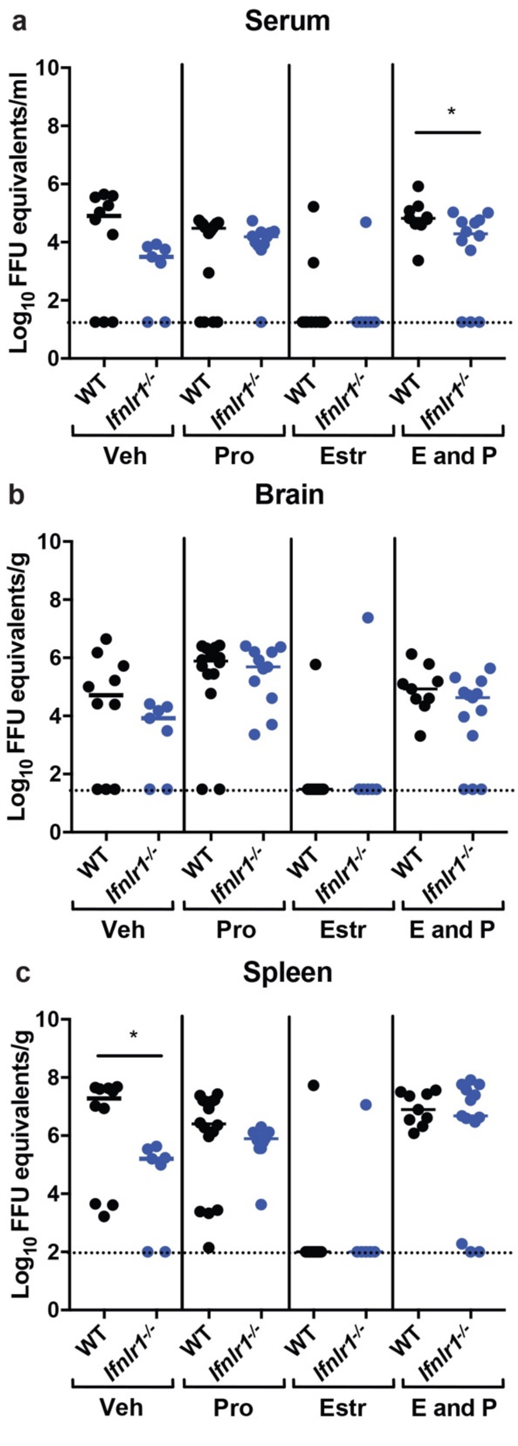

Supplementary Figure 5. Systemic spread of ZIKV in WT and *Ifnlr*$^{-/-}$ OVX mice after intravaginal infection. Six week-old WT or *Ifnlr1*$^{-/-}$ OVX mice were given a hormone replacement regimen of estradiol, progesterone, estradiol and progesterone, or vehicle. Mice were treated with 1 mg of anti-Ifnar1 mAb at day -1. On day 0 mice were inoculated via intravaginal

route with 10^6 FFU of ZIKV. At 7 dpi, ZIKV RNA was measured from the serum (**a**), brain (**b**), and spleen (**c**). Dotted lines indicate the LOD. Results are pooled from two or three experiments. Bars indicate median values (Mann-Whitney test: *, $P < 0.05$). Numbers of mice: Vehicle: WT + anti-Ifnar1, n = 10; *Ifnlr1*$^{-/-}$ + anti-Ifnar1, n = 7. Progesterone: WT + anti-Ifnar1, n = 16; *Ifnlr1*$^{-/-}$ + anti-Ifnar1, n = 11. Estradiol: WT + anti-Ifnar1, n = 10; *Ifnlr1*$^{-/-}$ + anti-Ifnar1, n = 6. Estradiol and progesterone: WT + anti-Ifnar1, n = 9; *Ifnlr1*$^{-/-}$ + anti-Ifnar1, n = 13.

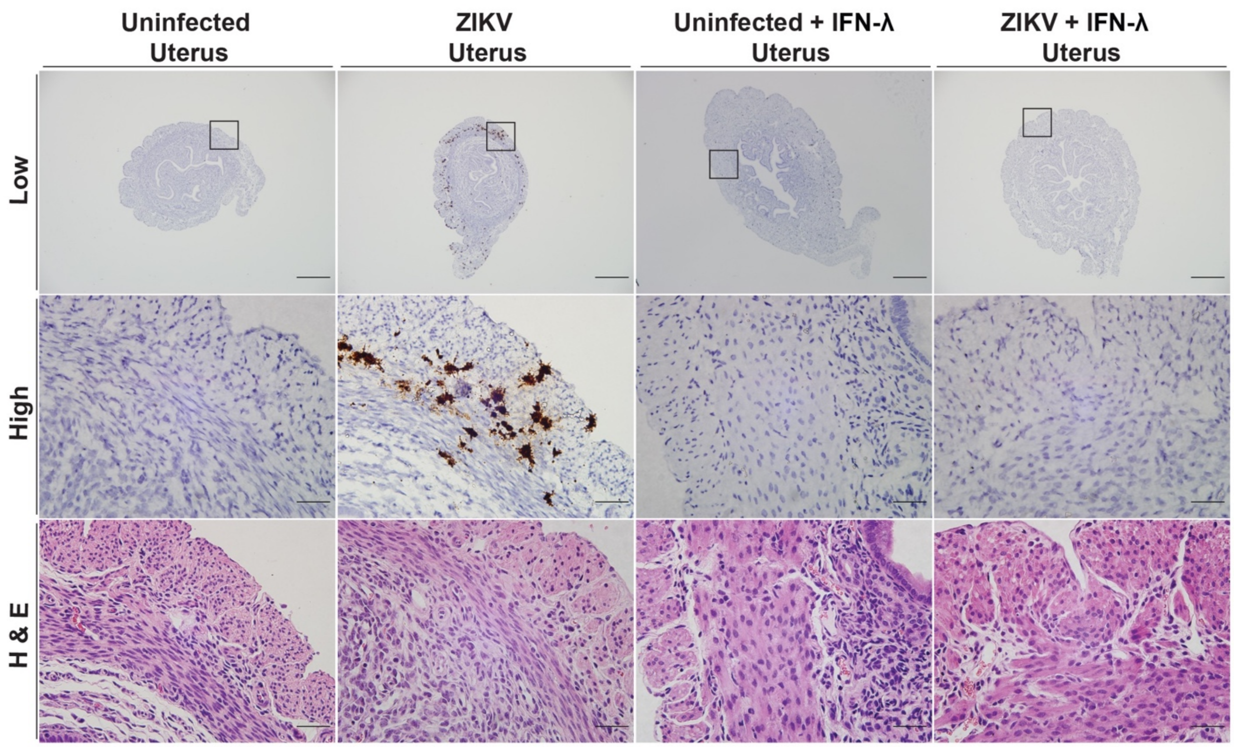

Supplementary Figure 6. Pegylated IFN-λ2 protects the uterus after intravaginal ZIKV infection in estradiol and progesterone treated OVX WT mice. Six week-old WT OVX mice were given a hormone replacement regimen of estradiol and progesterone. Mice were treated with 1 mg of anti-Ifnar1 mAb at day -1. On day 0 mice were treated via intravaginal route with 25 μg of pegylated IFN-λ2. Eight hours later, mice were inoculated with 10^6 FFU of ZIKV (Dakar 41525). At 7 dpi, ZIKV RNA was stained in uterine tissue using ISH. Results are pooled from two or three experiments. Low magnification scale bar = 500 μm, high magnification scale bar = 50 μm. Numbers of mice: WT + anti-Ifnar1, IFN-λ2, n = 9; WT + anti-Ifnar1, n = 10. Representative images are shown.
