# Supplementary material for: Interferon lambda protects the female reproductive tract against Zika virus infection
Source: Nat Commun. 2019 Jan 17;10:280. doi: 10.1038/s41467-018-07993-2 (PMC6336786; doi:10.1038/s41467-018-07993-2)
Supplement: Supplementary file 2 — Reporting Summary [file 41467_2018_7993_MOESM2_ESM.pdf]

nature research

Corresponding author(s): Michael Diamond

Reporting Summary

Nature Research wishes to improve the reproducibility of the work that we publish. This form provides structure for consistency and transparency in reporting. For further information on Nature Research policies, see Authors & Referees and the Editorial Policy Checklist.

Statistical parameters

When statistical analyses are reported, confirm that the following items are present in the relevant location (e.g. figure legend, table legend, main text, or Methods section).

n/a	Confirmed	
☐	☒	The exact sample size (*n*) for each experimental group/condition, given as a discrete number and unit of measurement
☐	☒	An indication of whether measurements were taken from distinct samples or whether the same sample was measured repeatedly
☐	☒	The statistical test(s) used AND whether they are one- or two-sided<br>*Only common tests should be described solely by name; describe more complex techniques in the Methods section.*
☒	☐	A description of all covariates tested
☐	☒	A description of any assumptions or corrections, such as tests of normality and adjustment for multiple comparisons
☐	☒	A full description of the statistics including central tendency (e.g. means) or other basic estimates (e.g. regression coefficient) AND variation (e.g. standard deviation) or associated estimates of uncertainty (e.g. confidence intervals)
☒	☐	For null hypothesis testing, the test statistic (e.g. *F*, *t*, *r*) with confidence intervals, effect sizes, degrees of freedom and *P* value noted<br>*Give P values as exact values whenever suitable.*
☒	☐	For Bayesian analysis, information on the choice of priors and Markov chain Monte Carlo settings
☒	☐	For hierarchical and complex designs, identification of the appropriate level for tests and full reporting of outcomes
☒	☐	Estimates of effect sizes (e.g. Cohen's *d*, Pearson's *r*), indicating how they were calculated
☐	☒	Clearly defined error bars<br>*State explicitly what error bars represent (e.g. SD, SE, CI)*

Our web collection on statistics for biologists may be useful.

Software and code

Policy information about availability of computer code

Data collection	No software used
Data analysis	GraphPad Prism 7.0, CLC Genomics Workbench 9.0, DESeq2 package in R, Gene set enrichment analysis (GSEA) v2.0, Sailfish (version 0.6.3)

For manuscripts utilizing custom algorithms or software that are central to the research but not yet described in published literature, software must be made available to editors/reviewers upon request. We strongly encourage code deposition in a community repository (e.g. GitHub). See the Nature Research guidelines for submitting code & software for further information.

Data

Policy information about availability of data

All manuscripts must include a data availability statement. This statement should provide the following information, where applicable:

- Accession codes, unique identifiers, or web links for publicly available datasets
- A list of figures that have associated raw data
- A description of any restrictions on data availability

All datasets generated during and/or analyzed during the current study are available from the corresponding authors upon reasonable request.

Field-specific reporting

Please select the best fit for your research. If you are not sure, read the appropriate sections before making your selection.

☒ Life sciences ☐ Behavioural & social sciences ☐ Ecological, evolutionary & environmental sciences

For a reference copy of the document with all sections, see nature.com/authors/policies/ReportingSummary-flat.pdf

Life sciences study design

All studies must disclose on these points even when the disclosure is negative.

Sample size	We used a power calculation (80% power, 0.05 type I error) to see an 3 to 5-fold effect in vivo (depending on data distribution).
Data exclusions	No data were excluded
Replication	All cell culture experiments were repeated multiple independent times. In vivo experiments were performed with independent repeat experiments.
Randomization	There was no randomization of animals for this study.
Blinding	Not blinded. Although the study was not blinded, key experiments were repeated independently by multiple members of the laboratory

Reporting for specific materials, systems and methods

Materials & experimental systems

n/a	Involved in the study	
☐	☒	Unique biological materials
☐	☒	Antibodies
☐	☒	Eukaryotic cell lines
☒	☐	Palaeontology
☐	☒	Animals and other organisms
☒	☐	Human research participants

Methods

n/a	Involved in the study	
☒	☐	ChIP-seq
☒	☐	Flow cytometry
☒	☐	MRI-based neuroimaging

Unique biological materials

Policy information about availability of materials

Obtaining unique materials	No restrictions. Human primary vaginal and cervical epithelial cells are available commercially. WT and KO mice are available commercially or via an MTA

Antibodies

Antibodies used	Anti-Ifnar1
Validation	Commercial (Leinco). Binds to IFNAR1 via flow cytometry. Blocks IFN signaling as judged by a bioassay

Eukaryotic cell lines

Policy information about cell lines

Cell line source(s)	Vero cells (ATCC)
Authentication	Cells were purchased from ATCC
Mycoplasma contamination	Tested monthly and judged free of mycoplasma contamination using a commercial kit

Commonly misidentified lines (See ICLAC register)	*Name any commonly misidentified cell lines used in the study and provide a rationale for their use.*

Animals and other organisms

Policy information about studies involving animals; ARRIVE guidelines recommended for reporting animal research

Laboratory animals	WT and hu-STAT2 C57BL6 mice; congenic Ifnar1-/- or Ifnlr1-/-
Wild animals	N/A
Field-collected samples	N/A
